# Case Study of Capacity Building for Smoke-Free Indoor Air in Two Rural Wisconsin Communities

**Published:** 2007-09-15

**Authors:** Shelly Mahon, Ellen Taylor-Powell

**Affiliations:** University of Wisconsin–Madison, Human Development and Family Studies. Ms Mahon is a doctoral candidate at the University of Wisconsin–Madison; University of Wisconsin–Extension, Madison, Wisconsin

## Abstract

**Background:**

Despite national declines in smoking prevalence, disparities that pose challenges to tobacco control efforts exist among rural manufacturing populations. This community case study sought to better understand the dynamics and nuances that facilitate or impede capacity-building efforts in rural communities.

**Context:**

Two rural manufacturing communities in Wisconsin with similar demographic characteristics were chosen for study. One represented farming communities with close proximity to a metropolitan area, and the other represented more isolated communities.

**Methods:**

The qualitative case study used a collaborative approach to collect data in four areas of research: 1) community context, 2) coalition functioning, 3) partnerships, and 4) strategy implementation. Data were analyzed using standard content analysis and triangulated for clarity and consistency.

**Consequences:**

Although not all the factors found to influence capacity-building efforts were unique to rural environments, the effects were impacted by rural isolation, small population sizes, local attitudes and beliefs, and lack of diversity and resources. Differences in coalition leadership and strategy implementation influenced the effectiveness of the capacity-building efforts in each community, bringing attention to the unique nature of individual contexts.

**Interpretation:**

Implementing capacity-building efforts in rural communities requires skilled and dedicated local leaders who have ready access to training and support (i.e., technical, emotional, and financial). Pairing of rural communities with greater use of distance technologies offers a cost-effective approach to reduce isolation and the constraints of financial and human resources.

## Background

Despite a national decline in adult smoking prevalence, tobacco-use burden in rural communities is evident among people of the working class, with low levels of education, and with low income and is prevalent among members of all races/ethnicities, both sexes, and all age groups (1). Other socioeconomic variables related to health protection disparities include geographic location, voter support for state cigarette tax initiatives, and board of health funding. If not considered, these variables actually foster disparities in health protection and undermine the *Healthy People 2010* goal of eliminating health disparities (2). A recent Wisconsin study of 15 communities engaged in campaigns to pass smoke-free restaurant ordinances during 1992–2002 found successful campaigns linked with areas of higher adjusted gross income, with a greater percentage of Democratic voters, and with higher smoking rates, as well as areas using coalitions with previous political experience, identified grassroots supporters, and extensive media coverage, including editorial support of local newspapers (3). With smoking prevalence among blue-collar workers nearly double that of white-collar workers in 1997, rural communities represent Wisconsin's next tier of communities to be reached (4). This study was funded by the American Legacy Foundation through a contract with Smoke Free Wisconsin (SFW), the principal tobacco advocacy resource in Wisconsin. The purpose of this study was to provide an in-depth perspective of the factors that facilitate or impede smoke-free indoor air capacity-building in two rural Wisconsin communities.

## Context

In 2005, SFW requested and received funds of $100,000 from the American Legacy Foundation, Small Innovative Grants Program, to examine capacity-building strategies for smoke-free indoor air in rural environments. Formed in 2000, SFW is a single-focus organization committed to reducing tobacco use in Wisconsin through policy change. SFW contracted with the University of Wisconsin–Extension (UW–Extension) to  conduct a case study evaluation in two rural Wisconsin manufacturing communities in order to better understand what strategies appear to be more successful in getting communities ready for smoke-free indoor air campaigns. The American Legacy Foundation, SFW, the UW–Extension, and the two participating communities collaborated throughout the study.

The two communities selected for the case study represented two types of rural environments in Wisconsin. Community 1 represented rural communities with farmland and more access to metropolitan cities. Community 2 represented more isolated communities located in the northern part of the state. Both communities were committed to addressing local disparities by targeting those with blue-collar jobs, low education levels, and low income. Each community had a population of approximately 10,000, was designated as *rural* by the U.S. Census Bureau, and had more than 20% of its jobs in manufacturing (5).

## Methods

Using a participatory approach, collaborators identified four areas to guide data collection during the 9-month period from August 2005 through April 2006. These included 1) community context, 2) coalition functioning, 3) partnerships, and 4) strategy implementation. The Institutional Review Board of the UW–Extension provided a review and subsequent approval for the study. Data collection involved multiple sources including 1) documents and historical records, 2) pre- and post-readiness assessment surveys, 3) teleconference calls and meetings initiated by SFW, 4) biweekly telephone conversations between the evaluator and coalition coordinators, 5) observations at coalition meetings and special events, 6) community site visits, 7) key informant interviews (i.e., with coalition coordinators, coalition members, key influentials [e.g., restaurant/bar owners, business owners, school officials, politicians], and community leaders), and 8) ad hoc telephone and e-mail dialogues.

Data were entered into NVivo 7 (QSR International Pty Ltd, Cambridge, Massachusetts) software in order to handle the rich text–based information. An analysis process outlined by grounded theorists was used (6), interview transcripts were coded, and observational field notes were open coded (i.e., line-by-line analysis) then coded axially (i.e., focused coding). Coding, analysis, and interpretation continued over the course of the study period, ensuring that all data were triangulated and compared for clarity and consistency. 

## Consequences

### Community context

Demographic characteristics of the participating communities are presented in the Table. Although both communities were defined as *rural*, the two differed in significant ways. Community 1 was wealthier, younger, better educated, more Democratic, and located near a major metropolitan area that provided a daily paper and media outlets. As a tourist and recreational destination, it had experienced an influx of outsiders, diverse perspectives, and significant growth since 2000. In contrast, community 2 was less diversified, less economically vibrant, and located in a more isolated part of the state that received only weekly news coverage or daily papers from neighboring towns. This community had experienced a decline in both economy and population since 2002. Other community factors impacting capacity-building efforts included events in neighboring locales, population size, social rules, and local attitudes.

The perceived loss of business resulting from media coverage of controversies over smoking bans in bordering towns created heightened tension and a tendency for residents to favor a county or statewide ban. As a member from community 1 expressed, "The effort to target bars and restaurants is what makes it difficult. People are afraid of losing business. I am not eager for the people who do business in our town to be at an economic disadvantage to people who live in the surrounding communities." Furthermore, small populations in rural settings meant members performed multiple roles in multiple groups. A participant from community 2 explained, "You don't get a lot of diverse public support in rural communities because the leaders are often involved in more than one network." This participation by community leaders in multiple roles resulted in irregular attendance at meetings and only a few individuals willing and available to engage in strategy implementation. Related to size was the phenomenon that "everyone knows everything" in rural settings. "You have to 'make nice' because everything you say will spread very fast and come right back to bite you. This is a social rule in rural communities." Individuals also feared that public knowledge of their position would result in loss of business or public support (i.e., votes), contributing to an unwillingness to be connected with readiness efforts.

A dominant issue in both communities was the debate about personal rights and government intervention. This issue was probably exacerbated during our study by the pervasive news coverage related to the war on terror, gay marriage, illegal immigration, global warming, and the government's ability to govern. As a respondent in community 1 stated, "People in rural communities have been fighting governmental control over things such as hunting and fishing for years. People already have a mentality of 'We don't want government involved in our decisions.' Therefore, government regulation of other issues immediately puts people on the defens[ive]." Many held the belief that individuals chose to work in, or frequent, establishments that allow smoking and could "go someplace else" if they did not like smoke. However, participant comments suggested that residents were limited in their options for employment and dining out:

People think that those who do not want to work in restaurants or bars that allow smoking can go work someplace else. However, there are not a lot of jobs in rural communities. Entry-level positions for people with very few skills and little education tend to be in places that allow smoking. We need public awareness that these disparities limit where people can work.

### Coalition functioning

**History**

Coalitions are rooted in the community, providing opportunities to pool resources, abilities, and expertise to address community issues (7-12). Although both communities had existing coalitions, one began operating in the early 1990s through Project Assist and the other was formed in 2000. Each used financial resources differently depending on its experience, community context, existing structure, and operational capacity. Community 1 relied on an existing coalition with the coalition coordinator as lead, while community 2 hired a part-time leader to develop a core group to implement the capacity-building work that was funded under the grant to promote smoke-free indoor air.

**Leadership**

Our research supported existing literature related to the important role of leadership in community capacity building. Both leaders had "townie" status, meaning they were known and accepted as residents of the community. As one leader shared, "Those who are not *of* the rural community can buy their way into the country club, but that doesn't buy their way into influence and trust." Townie status entitled them to local knowledge, credibility, acceptance, and connections to the media, policy makers, and other community networks. However, the planning and implementation of strategies in each site were influenced by differences in leader personalities, interests, skills, and experience in 1) tobacco control, 2) coalition/group development and management, and 3) community organizing. The leader from community 2 could be characterized as task-focused and the leader from community 1 as process-focused. The task-focused leader was hired to accomplish a set of activities during the grant period. In contrast, the process-focused leader had an ongoing position as coalition leader for the previous 12 years. With little previous experience, the task-focused leader relied on public health staff for support. The process-focused leader worked toward continued outreach and sustainability through teamwork, shared decision-making, and coalition building to implement the smoke-free indoor air strategies.

**Functioning**

The coalition's level of functioning was largely tied to its membership base and financial resources. The leader in community 1 stressed the importance of having members with complementary expertise and skills and at least one member who could foster involvement and community change: "I need someone who can help with education and political organizing, someone who has the chutzpah or courage to persist, to push people. I do not push hard enough. I want to partner with someone who has thicker skin." The leader in community 2, who had a more limited role, relied largely on herself, the public health department staff, and a few members who volunteered for specific tasks.

Access to funding with local discretion in use, based on jointly determined accountability standards, facilitated community-based strategy implementation in community 1. The leader said, "My role is to make sure the coalition continues to be funded." This coalition worked out of a unique funding structure that paid the coordinator as an independent contractor and allocated extra funds to engage, reward, and reimburse others for tobacco control activities. This structure also boosted involvement, distributed ownership, cemented commitment to smoke-free indoor air, spread visibility, and ultimately helped the coalition accomplish its objectives. Hired under the grant, the leader in community 2 faced the ticking clock of the grant timeline. This community moved from asking individuals to participate to encouraging individual sign-up and commitment to specific coalition activities.

### Partnerships

Partnerships provide access to resources such as expertise, money, influence, and individuals' power to facilitate goal achievement. Although partnerships with local, regional, state, and national entities were an expected part of the capacity-building work in both communities, both sites appeared limited to two primary partnerships. The principal partnership was with each community's department of public health as a function of funding, responsibility, and accountability. This partnership provided the coalitions with varying levels of technical assistance, financial and logistical resources, and emotional support.

A second partnership between the two participating sites emerged as a result of the research project. Biweekly teleconference calls, facilitated by the project evaluator, allowed the two leaders to share information and access external resources and expertise. Individuals at both sites expressed interest in building collaborative relationships with other rural coalitions to reduce isolation and increase learning from the successes and mistakes of others. As one of the leaders stated:

I hope this research reflects the benefit of pairing two rural communities and having an outside person to facilitate regular discussions. I found that the smallest ideas sparked bigger ideas. Having regular dialogue about strategies and sharing resources with each other was helpful and cost-effective. This breaks down the feeling of rural isolation.

### Smoke-free indoor air strategies implemented

**Community education**

Both communities viewed education as an important and ongoing process for building smoke-free indoor air capacity. Principal activities included 1) implementation of Family and Community Town Suppers (FACT Suppers); 2) development and distribution of newsletters, brochures, and other educational materials; 3) communication through e-mail lists, media releases, and individual discussions; and 4) implementation of presentations and exhibits. The FACT suppers proved difficult to plan and implement given the time and effort required. Community 1 preferred monthly e-mail and hard-copy newsletters because of the perceived reach and ability to affect attitudes of these two media.

Being able to see information about smoke-free policies in print helps people to see that it is okay to ask for a smoke-free indoor air ordinance. . . . The e-newsletter is a powerful tool for reaching individuals in rural communities who might be isolated from receiving information about environmental tobacco smoke.

Community 2 relied heavily on educational posters that conveyed messages stressing the health risks associated with secondhand smoke.

**Mobilizing support**

Communities mobilized support of individuals, organizations, and leaders through various efforts including 1) using newspaper inserts; 2) using signature pledge sheets and collecting petition signatures; 3) displaying pledges/petitions at select locations; 4) placing telephone calls; 5) mining e-mail lists to identify potential supporters; 6) contacting individuals, local organizations, clubs, businesses, and labor leaders; 7) soliciting at community events and meetings; and 8) maintaining a database of supporters. In both communities, networking was an important strategy. The leader in community 1 described the distinction between networking in rural and urban settings:

I can draw on my experience as an organizer in urban and rural communities for a comparison. In an urban community, I contacted the people who held the reins of larger organizations. I connected with these people very quickly on the basis of cognitive content. Once I determined who my allies were, I called the heads of these groups together to establish some semblance of trust and cooperation. The smaller group of leaders represented hundreds of people from multiple organizations all linked together. However, in rural communities there are not a lot of well-established networks. The best public strategy I have found is to follow threads using a community connections map. I find an ally, educate that ally about the work that the coalition is doing, and then have that person contact everyone they know.

Although community 1 used a "community connections map" (i.e., individuals are asked to identify other potential supporters, and connections grow outward) to identify and build a list of potential supporters, community 2 relied heavily on signature sheets. Being "nice," personal, culturally appropriate, nonconfrontational, and meeting individuals "where they are" were highlighted as important qualities to succeed at networking. In the words of one respondent, "You cannot burn bridges in rural communities. Burning one bridge can sour several other community relationships."

**City council support**

To understand and gain city council support, the two communities engaged in various activities such as 1) tracking decision making and voting patterns, 2) increasing community members' familiarity with local government, 3) assessing individual members' level of support, and 4) identifying potential supporters, including a champion. Both coalitions stressed the importance of having members or outside supporters who could effectively communicate with the council. Community 2 implemented a community telephone survey in order to generate "hard data" considered necessary for gaining council support.  

**Reaching local unions**

The two communities took different approaches to reaching local unions, neither of which proved successful. In proximity to a metropolitan area, community 1 accessed a city-based union representative only to find he was largely unavailable and unengaged in local issues. Community 2 attempted to reach factory workers through the human resource staff and an industrial advisory committee that only resulted in the placement of onsite educational posters. The leader from community 2 stressed the need for more creative strategies and "impact-loaded" messages:  "Factory workers are well aware of the health hazards of second-hand smoke. Discussions around the cost of health care and loss of productivity to employers may be more effective messages."

**Media presence**

Media presence in both communities came primarily from newspaper coverage including 1) assessing the level of support from media staff, 2) working directly with reporters, 3) writing letters to the editor, and 4) submitting regular monthly articles to the newspaper or church bulletins. Despite an ambitious media plan, community 1 faced a lack of support among a newspaper staff thought to have Libertarian views and had to compete with coverage of a school referendum that captured media attention. As a result, the coordinator focused attention on monitoring and sharing tobacco-related news with members and supporters. The two local weekly newspapers in community 2 were more supportive, publishing seven of the eight articles submitted, with several gaining prime location in the local papers.

## Interpretation

Although the factors influencing these rural capacity-building efforts did not always differ from factors in urban environments, issues of rural isolation, small population sizes, limited resources, conservative attitudes, and limited diversity intensified the effects. Attitudes about smoking and smoking bans represented a complex interplay of personal beliefs and contextual factors that ultimately had an impact on capacity-building efforts. Differences in coalition leadership and variations in leaders' experiences and history with tobacco control efforts, community organizing, and coalition management were major factors influencing strategy decision-making and implementation. Combining findings relevant to both rural and urban communities with findings more specific to rural settings illustrates the complexity of capacity-building efforts for smoke-free indoor air. These results may be especially useful to newer members and groups, as well as to statewide groups providing training and technical assistance to support policy change for smoke-free indoor air. Results specific to rural communities add to existing literature, providing a better understanding of effective ways to generate support for public health issues. It is expected that the findings have application beyond tobacco control to other community-based public health capacity-building efforts.

- Like urban communities, rural communities are not homogeneous. Important contextual factors vary that directly influence capacity-building work. Ongoing monitoring and adaptations to a dynamic local environment require time, skill, and dedication.
- Not all factors affecting capacity-building efforts in rural environments are unique to the rural milieu. 
- Capacity building is largely a function of the skills, personality, vision, and experience of local leaders. Support and training of coalition leaders is important, especially in rural communities that otherwise may be isolated and limited in human, technical, and financial resources.
- Financial resources and budgetary arrangements are key drivers. Adequate funds with local discretion in use, based on jointly determined accountability standards, facilitate community-based strategy implementation.
- Expanded partnerships, sharing across rural coalitions, and having ready access to evidence-based practices are crucial for capacity-building efforts. A model based on the pairing of rural communities and greater use of distance technologies to connect dispersed coalitions could be a cost-effective strategy to enhance desired outcomes. 
- Training, sharing, and learning among seasoned leaders and national and international experiences benefit capacity building. This sharing might take the form of a "buddy" system of learning partners or a "learning community" comprising interested tobacco-free coalitions.
- Single-purpose or resource-intensive strategies are less likely to be implemented. Rural coalitions favor strategies that achieve multiple objectives and produce a high return for their investment.
- Working with elected officials demands particular abilities and skills that need to be present or developed in coalition members or community supporters.
- Having limited resources (e.g., time, money, person-power) affects what is undertaken and the extent to which rural communities are able to move toward community readiness. Influential, involved members often assume multiple roles in rural communities, affecting both how and to what extent members are able to engage.
- More creative, relevant, and impact-loaded educational messages are needed if unions or blue-collar groups are to be targets for policy change efforts for smoke-free indoor air. 
- Social interactions, relationships, and network building take time, have long-term consequences, and take on added significance in rural communities.

## Figures and Tables

**Table:** Demographic Characteristics of Two Rural Communities, Wisconsin, 2005–2006^a^

Characteristic	Community 1	Community 2
Population	10,711	10,146
Median household income, $	38,375	33,098
Per capita income, $	19,304	17,429
Mean age, y	35.8	37.3
Closest metro city, miles	40	190
**Race/ethnicity, %**		
White	96.2	97.3
Hispanic	1.6	1.0
African American	0.5	0.2
American Indian	1.0	0.8
Two or more races	0.7	0.7
**Political affiliation, %**		
Democrat	51.6	47.7
Republican	47.4	51.1
Unknown	1.0	1.2
**Educational attainment, %**		
<Graduate/professional degree	94.3	94.8
≥Graduate/professional degree	5.7	5.2
**Religious affiliation, %**		
Religious	65.0	69.0
Not religious	35.0	31.0

a Data were derived from the 2000 U.S. Census (13).
